# Characterization of Developmental Pathway of Natural Killer Cells from Embryonic Stem Cells In Vitro

**DOI:** 10.1371/journal.pone.0000232

**Published:** 2007-02-21

**Authors:** Nooshin Tabatabaei-Zavareh, Anastasia Vlasova, Chelsea Pamela Greenwood, Fumio Takei

**Affiliations:** Terry Fox Laboratory, British Columbia Cancer Research Center and Department of Pathology and Laboratory Medicine, University of British Columbia, Vancouver, British Columbia, Canada; New York University School of Medicine, United States of America

## Abstract

In vitro differentiation of embryonic stem (ES) cells is often used to study hematopoiesis. However, the differentiation pathway of lymphocytes, in particular natural killer (NK) cells, from ES cells is still unclear. Here, we used a multi-step in vitro ES cell differentiation system to study lymphocyte development from ES cells, and to characterize NK developmental intermediates. We generated embryoid bodies (EBs) from ES cells, isolated CD34^+^ EB cells and cultured them on OP9 stroma with a cocktail of cytokines to generate cells we termed ES-derived hematopoietic progenitors (ES-HPs). EB cell subsets, as well as ES-HPs derived from EBs, were tested for NK, T, B and myeloid lineage potentials using lineage specific cultures. ES-HPs derived from CD34^+^ EBs differentiated into NK cells when cultured on OP9 stroma with IL-2 and IL-15, and into T cells on Delta-like 1-transduced OP9 (OP9-DL1) with IL-7 and Flt3-L. Among CD34^+^ EB cells, NK and T cell potentials were detected in a CD45^−^ subset, whereas CD45^+^ EB cells had myeloid but not lymphoid potentials. Limiting dilution analysis of ES-HPs generated from CD34^+^CD45^−^ EB cells showed that CD45^+^Mac-1^−^Ter119^−^ ES-HPs are highly enriched for NK progenitors, but they also have T, B and myeloid potentials. We concluded that CD45^−^CD34^+^ EB cells have lymphoid potential, and they differentiate into more mature CD45^+^Lin^−^ hematopoietic progenitors that have lymphoid and myeloid potential. NK progenitors among ES-HPs are CD122^−^ and they rapidly acquire CD122 as they differentiate along the NK lineage.

## Introduction

Natural killer (NK) cells are a lymphocyte population that plays an important role in the innate immune system. They are characterized by their natural cytotoxicity against tumor cells and virus-infected cells, but they are also an important source of cytokines. Unlike T and B cells, NK cells do not express antigen-specific receptors generated by the somatic rearrangement of receptor genes. Instead, they express various combinations of inhibitory and stimulating receptors to recognize a broad range of target cells [1]. NK lineage committed precursors (NKPs) that differentiate into NK cells but not other hematopoietic cells have been identified in adult mouse bone marrow (BM) by the surface phenotype of Lin^−^CD122 (IL-2Rβ)^+^ [2], and the developmental processes from NKPs to mature NK cells have been described in detail [3]. On the other hand, the developmental pathway from hematopoietic stem cells (HSCs) to NKPs is still unclear. It is generally thought that all lymphocytes derive from common lymphoid progenitors (CLPs) identified by the surface phenotype of Lin^−^c-kit^lo^Sca-1^lo^IL-7Rα^+^ in BM [4]. However, bipotent T/NK progenitors in fetal liver, blood and thymus give rise to both NK and T cells but not B cells [5]–[7]. Some fetal T/NKP progenitors are also identified as IL-7R^+^ [6], [8], suggesting that the expression of IL-7R is a critical stage in lymphocyte development in both adult and fetal environment.

In vitro differentiation of embryonic stem (ES) cells provides a powerful model system to study lymphocyte development from hematopoietic progenitors. Embryoid bodies (EBs) generated from ES cells in vitro contain CD34^+^ cells that have both myeloid and lymphoid potential [9]. B cells [9]–[12], NK cells [9], and T cells [13], [14] have also been generated from ES cells in vitro. In most studies, ES cells were co-cultured on OP9 stromal cell line to generate cells of the hematopoietic lineage [10], [12], [13]. However, the differentiation pathways from ES cells to lymphocytes and the intermediate progenitors have not been characterized in detail. It is important to isolate hematopoietic precursors with lymphoid potential from ES cells, because lymphomyeloid precursors from ES cells could be used as the potential source of reconstituting HSCs in the treatment for leukemia and a range of genetic disorders.

We have previously established a multi-step culture system to induce ES cell differentiation into NK cells. In this system, ES cells were induced to form EBs, and CD34^+^ hematopoietic progenitors isolated from EBs were cultured with OP9 and cytokines for differentiation into ES-derived hematopoietic progenitors (ES-HPs), which subsequently differentiate into NK cells [15]. No other lymphocytes are generated in this culture system. Here, we used this ES culture system to characterize NK cell progenitors at different steps in development and examined the relationship between NK and other lymphoid lineages.

## Materials and Methods

### Cell lines, antibodies and flow cytometry

OP9 cells were obtained from RIKEN (Tokyo, Japan). OP9 cells transduced with Delta-like1 and green fluorescent protein (OP9-DL1) were kind gift from Dr. J-C Zuniga Pflucker (Toronto, Canada). 2.4G2 (anti-FcRγ), FITC-conjugated and purified anti-mouse Ly5.2 (CD45.2) and Ly5.1, biotinylated anti-Mac-1 (M1/70), and Gr-1 (RB6-8C5) mAbs have been described [16], [17]. Biotinylated anti-CD34 (RAM34), and IL-7Rα (A7R34) mAbs were purchased from eBioscience (San Diego, CA). FITC-conjugated anti-CD34 (RAM34), purified anti-CD31 (MEC13.3), phycoerythrin (PE)-conjugated anti-CD25 (3C7), IL-2Rβ (TM-β1), and Sca-1 (E13-161.7) mAbs, biotinylated antibodies to mouse c-kit (2B8), CD31 (MEC-13.3), common γ-chain (γc) (TUGm2), CD94 (18d3), NKG2A/C/E (20d5), TCRγδ (GL3), CD3ε (145-2C11), CD44 (1M7), CD4 (GK1.5), Ter119, B220 (RA3-6B2), CD19 (1D3), streptavidin-APC, -PE, -FITC, and proper isotype control antibodies were purchased from BD-Biosciences (Mississauga, ON). Biotinylated anti-mouse TCRαβ (H57-597) mAb was purchased from Cedarlane (Ontario, Canada). PE-conjugated anti-mouse CD8α (53-6.7) mAb was purchased from Boehringer Mannheim Biochemica. Secondary antibodies goat anti-mouse IgG and goat anti-rat IgG labeled with Alexa Flour 647 were obtained from Molecular Probes (Eugene, OR). For all cell stainings and sortings, cells were first pre-incubated with 2.4G2 hybridom supernatant or human γ globulin (Sigma-Aldrich) to block Fc-receptors, followed by primary mAbs. All incubations were performed on ice for 30 min and stained cells were analyzed on a FACSCalibur (BD Biosciences, San Jose, CA) with the CellQuest Pro software (BD Biosciences). Cell sorting was carried out on a FACSVantage^TM^ SE (BD Biosciences).

### ES cell culture

The ES cell line R1 (129/SvJ strain) was maintained as described [15]. On the day of differentiation, cells were harvested, resuspended at 1,500–2,000 cells/ml in the differentiation medium consisting of IMDM, 15% FBS, 1% methylcellulose, 2 mM L-glutamine, 150 µM MTG, 50 ng/ml mouse stem cell factor (SCF), 30 ng/ml mouse IL-3, and 30 ng/ml human IL-6, and 1 ml of the cell suspension was dispensed into 35 mm petri dish (StemCell Technologies, Vancouver, Canada). The cells were incubated at 37°C and 5% CO_2_ for 8 days for EB formation. EBs were then harvested, trypsinized and made into single-cell suspension by passing them through a 21-gauge 1½-inch needle three times. Subsequently, the cells were stained with biotinylated anti-CD34 followed by streptavidin-PE and FITC conjugated anti-CD45.2 or purified CD45.2 followed by Alexa Flour 647-conjugated goat anti-mouse IgG, and sorted on FACSVantage^TM^ SE (BD, San Jose, CA) for isolation of CD34^+^, CD34^+^CD45^−^ and CD34^+^CD45^+^ EB cells. EB cells were then seeded onto OP9 stroma in 24-well plates at a concentration of 4×10^4^ cells/well and cultured for 7 days with 30 ng/ml IL-6, 4 ng/ml IL-7, 40 ng/ml SCF, and 100 ng/ml Flt3-Ligand (Flt3-L) (Stem Cell Technologies) to generate ES-HPs. After the first 3–4 days of incubation, half the media was replaced with fresh medium containing the same cytokines. ES-HPs were harvested on day 7 by vigorous pipetting, washed, and passed through a 45 µm filter. Cells were then transferred onto lineage-specific cultures or stained for FACS analysis.

### Lineage differentiation cultures

OP9 and OP9-DL1 cells were incubated for 2 days in 24 well plates to pre-form stroma layers. For NK differentiation cultures, EB cells or ES-HPs were plated on OP9 layers in OP9 media (αMEM supplemented with 10% FBS) containing 1,000 U/ml IL-2 (PeproTech, Rock Hill, NJ) and 5 ng/ml IL-15 (StemCell Technologies). Cells were cultured for 7–14 days before FACS analysis. For T cell culture, EB or ES-HP cells were seeded onto OP9-DL1 stroma with 5 ng/ml IL-7, and 10 ng/ml Flt3-L and cultured for 8–14 days. After this period, media was removed from the culture and replaced with media containing Flt3-L and 1 ng/ml IL-7. Cells were cultured for additional 3–6 days and harvested for FACS analysis or RNA extraction. For B cell culture, ES-HP cells were cultured on OP9 stroma with 5 ng/ml IL-7 and 10 ng/ml Flt3-L for one week. In all of these cultures, stromal cells were replaced with new cells if the culture period was longer than one week. To test the myeloid colony forming capacity of EB or ES-HP cells, cells were resuspended in IMDM, transferred onto Methocult^TM^ GF M3434 media (StemCell Technologies) at the density of 1000/plate in duplicate and cultured for 7–14 days. The numbers of all colonies including erythroid burst-forming units (BFU-E), granulocyte-macrophage colonies (CFU-GM/CFU-G/CFU-M), and erythrocyte-containing mixed colony-forming units (CFU-GEMM) were scored.

### RT-PCR

RNA from bulk cells was isolated with QIAGEN's RNeasy® Mini Kit and reverse transcribed into cDNA using QIAGEN's Omniscript Reverse Transcription kit. For samples from limiting dilution cultures, cells in 96-well plates were lysed with 50 µl guanidinium isothiocyanate solution, RNA was isolated (as described [18]) and reverse transcribed using a 18-mer oligo (dT) primer (1 µg/µl) (New England BioLabs, Pickering, OT) and Superscript^TM^ II (Invitrogen, Carlsbad, CA). Aliquots (1/5) of cDNA thus generated were used in PCR reaction. Forward primers from TCR Vγ2, 3, 4, and 5, and reverse primer from the TCRγ constant region have been described [19]. The primer sequences were as follows: granzyme B (forward) 5′-CAAAGGCAGGGGAGATCATC-3′, (reverse) 5′-CTCTTCAGCTTTAGCAGCATG-3′; CD3ε (forward) 5′-GCCTCAGAAGCATGATAAGC-3′, (reverse) 5′-AGACTGCTCTCTGATTCAGG-3′; RAG-1 (forward) 5′-TGCAGACATTCTAGCACTCTGG-3′, (reverse) 5′-ACATCTGCCTTCACGTCGAT-3′; GAPDH (forward) 5′-TCAACGACCCCTTCATTGACCTC-3′, (reverse) 5′-AGACTCCACGACATACTCAGCAC-3′. The PCR thermocycling conditions for TCRγ RT-PCR were as follows: 5 min at 96°C followed by 40 cycles of 96°C for 15 s, 50°C for 40 s, 72°C for 1 min, and a final 10 min extension at 72°. The following condition was used for granzyme B RT-PCR: 1 min at 94°C followed by 40 cycles of 94°C for 30 s, 60°C for 30 s, and 72°C for 30 s. 18 µl of PCR products from bulk cultures were mixed with 2 µl of 10× loading buffer and analyzed on 1.2% agarose gel. The gel from TCRγ RT-PCR was alkaline blotted to ZetaProbe membranes (Bio-Rad, Hercules, CA). In limiting dilution experiments, the entire TCRγ PCR products from multiple samples were transferred onto ZetaProbe membranes using Bio-Dot® Microfiltration apparatus (Bio-Rad). The membrane was probed with biotin-labeled oligonucleotides and visualized by North2South® Chemiluminescent Nucleic Acid hybridization and Detection Kit (Pierce, Rockford, IL). The 3′ end of oligonucleotide was labeled with biotin-14-dATP according to Invitrogen protocol. The probe sequence was: Jγ1: 5′-TGCAAATACCTTGTGAAAACCTGAG-3′.

### Limiting dilution analysis

OP9 and OP9-DL1 cells were X-ray irradiated (30Gy) and pre-incubated for 2 days in 96 well plates. Limiting numbers of cells were deposited into 96-well plates using the CloneCyt Plus option of FACVantage^TM^ SE cell sorter. To determine NK progenitor frequency, cells were cultured for 10–12 days on pre-formed OP9 stroma in IL-2 and IL-15. For T progenitor frequency, cells were cultured on pre-formed OP9-DL1 stroma with 5 ng/ml IL-7 and 10 ng/ml Flt3-L for 10–12 days. Cells were then cultured with 1 ng/ml IL-7 for additional 3–5 days. After this culture period, RNA was isolated from cells as described in RT-PCR section. RNA thus generated was reverse transcribed into cDNA and used in PCR reaction. The presence of NK cells was confirmed by granzyme B RT-PCR (data not shown). To identify T cells by RT-PCR, a mixture of TCR Vγ2, 3, 4, and 5 primers was paired with a reverse primer from TCRγ constant region. Rearranged TCRγ transcripts were detected by southern blotting (see above and data not shown) [19]. Progenitor frequency was determined using L-Calc^TM^ software (StemCell Technologies).

## Results

### NK and T cell potentials of ES-HPs

**Figure 1:**
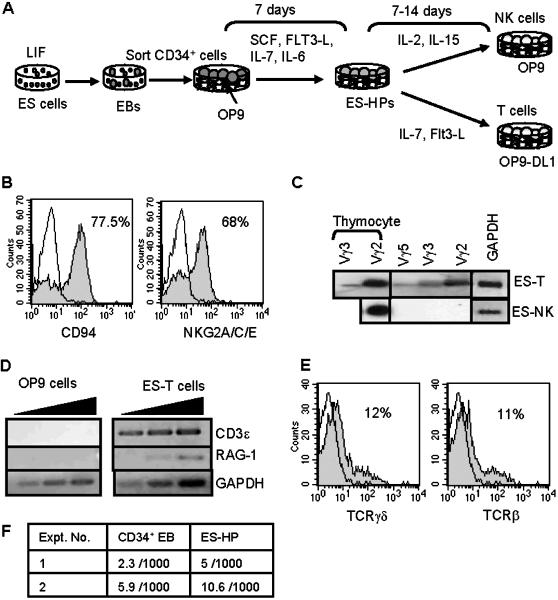
ES-HPs have NK and T cell potentials with little myeloid potential. (A) ES culture system to generate ES-HPs is illustrated. ES cells are induced to form EBs in methylcellulose. CD34^+^ EBs are sorted and cultured for one week with OP9 stroma in the presence of indicated cytokines to generate ES-HP cells. ES-HPs are cultured on OP9 stroma with IL-2 and IL-15 for NK cell differentiation or on OP9-DL1 stroma with IL-7 and Flt3-L for T cell differentiation. (B) NK cells generated from ES-HPs were stained for the indicated receptors and analyzed by flow cytometer. Filled histograms show staining with the appropriate mAbs and open histograms show isotype-matched control antibody staining. Percentages of positively stained cells over the control staining are shown. Dead cells were stained with propidium iodide and gated out. Residual OP9 cells were also gated out by their high scatter profile. (C) RNA was isolated from 2×10^5^ bulk T cells and 2×10^6^ bulk NK cells derived from ES-HPs (ES-T and ES-NK, respectively) and converted into cDNAs. Aliquots (1/30) of cDNAs were subjected to PCR for rearranged TCRγ gene. The PCR products were blotted and hybridized to a Jγ1 probe. Thymocytes were used as positive control. Glyceraldehyde-3-phosphate dehydrogenase (GAPDH) RT-PCR was also used as control. (D) cDNA was generated from ES-T cells as in (C). One microlitre of undiluted, 1/10 and 1/100 diluted cDNA was subjected to PCR for CD3ε and RAG-1. OP9 cells were used as negative control. (E) ES-T cells were harvested on day 7, stained for TCRγδ and TCRβ and analyzed by flow cytometer as in Fig. 1B. OP9-DL1 cells expressed green fluorescent protein and were gated out by green fluorescence. (F) One thousand CD34^+^ EBs and ES-HP cells were plated in methylcellulose media for myeloid and erythroid colony formation and the total number of myeloid and erythroid colonies were counted as described in materials and methods.

The previously described ES cell culture system [15] was slightly modified and used in this study (depicted in Fig. 1A). Briefly, R1 ES cells were cultured in methylcellulose for 8 days for EB formation. CD34^+^ EB cells were sorted and cultured on OP9 stromal cells with SCF, Flt3-L, IL-7 and IL-6 for 7 days. The cells generated in this culture, termed ES-HPs, further differentiated into NK cells upon co-culturing with OP9 in the presence of IL-2 and IL-15. The NK cells derived from ES cells (ES-NK cells) expressed CD94, NKG2A/C/E receptors on the surface as well as granzyme B transcipts (Fig. 1A, B and Table 1). We have previously shown that similar to fetal NK cells, ES-NK cells lack Ly49 expression but express NKRP1A (CD161) mRNA and are capable of differentially killing some tumor cell lines and MHC class I-deficient lymphoblasts [15]. To test whether ES-HPs have potential for the T cell lineage, they were co-cultured with OP9 stroma expressing Delta-like1 (OP9-DL1 cells), which promotes T cell differentiation from progenitors [20], in the presence of IL-7 and Flt3-L for 8–12 days. RT-PCR analysis of the resultant cells showed the expression of rearranged TCRγ mRNA (Fig. 1C) as well as CD3ε and RAG1 mRNA (Fig. 1D). Moreover, flow cytometric analysis showed the surface expression of TCRβ and TCRγ (Fig. 1E). Thus, ES-HPs included T cell progenitors. ES-HPs were also tested for the myeloid differentiation potential by in vitro colony forming cell assays. The ES-HP population contained very low frequency of erythroid/myeloid colony forming cells (Fig. 1F).

**Table 1:** Frequency of NK progenitors in EB and ES-HP subpopulations

Cells tested	NK progenitor frequency (% ± SD) †
CD34^+^CD45^−^ EB	0.16±0.03
CD45^+^ ES-HP*	1.9±1.2
CD45^−^ ES-HP*	0.35±0.05
Bulk ES-HP*	0.5#
CD45^+^Mac-1^−^Ter119^−^ ES-HP**	10±5.1
Bulk ES-HP**	0.4±0.15

† Progenitor frequency was determined by sorting limiting numbers of cells into 96 well plates containing OP9 stroma and media for NK differentiation cultures as described in materials and methods. NK cells were detected by granzyme B RT-PCR.

* Derived from CD34^+^ EB cells.

** Derived from CD34^+^CD45^−^ EB cells.

# Average of two independent experiments. All other values are average of three independent experiments.

**Figure 2:**
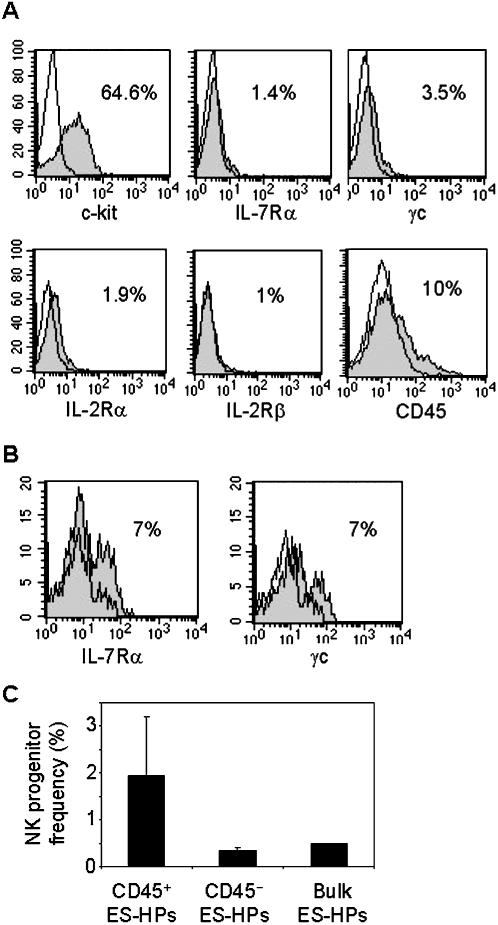
Characterization of NK progenitors within ES-HPs. (A) ES-HP cells were stained for the indicated markers and analyzed by flow cytometry. Filled histograms show staining with appropriate mAbs and open histograms show isotype-matched control antibody staining. Percentages of positively stained cells over the control staining are shown. Dead cells were stained with propidium iodide and gated out. OP9 cells were also gated out by their high scatter profile. (B) ES-HPs were co-stained with anti-CD45.2 and IL-7Rα mAbs (left panel), or anti-CD45.2 and γc mAbs (right panel) and analyzed by flow cytometer as in (A). CD45 positive cells were gated and analyzed for the expression of indicated receptors. (C) Irradiated OP9 cells were cultured for 2 days in 96 well plates. ES-HPs were stained with anti-CD45.2 mAb and CD45^+^ and CD45^−^ ES-HPs were sorted by FACS into the wells with the pre-formed OP9 stroma layers. For CD45^+^ ES-HPs, 10, 30 and 100 cells per well were sorted into 12 wells each. For CD45^−^ and bulk ES-HPs, 30, 100 and 300 cells per well were sorted into 12 wells each. After culturing with appropriate cytokines for NK cell differentiation, cells in individual wells were harvested and analyzed for the presence of NK cells by granzyme B RT-PCR. Statistical analysis was performed using L-Calc^TM^ software. The results are means ± SD of three independent experiments, except for bulk ES-HPs which is average of two experiments.

To further characterize lymphoid progenitors among the ES-HP population, we analyzed the expression of markers associated with BM lymphoid progenitors. Flow cytometric analysis showed that most ES-HPs expressed c-kit whereas only very small fractions expressed IL-7Rα (CD127), common γ chain (γc, CD132), IL-2Rα (CD25) and IL-2Rβ (CD122) (Fig. 2A). Furthermore, only 10% of ES-HPs expressed CD45, suggesting that the majority of the ES-HPs are non-hematopoietic or mature erythroid cells. As expected, cells expressing IL-7Rα and γc were enriched in the CD45^+^ subset of ES-HPs (Fig. 2B). Limiting dilution culture analysis for NK progenitors showed that on average 0.5% (1 in 202) of the bulk ES-HPs differentiate into NK cells. As expected, NK progenitors were more enriched in CD45^+^ ES-HPs (1 in 53 or 1.9%) than in CD45^−^ ES-HPs (1 in 286) (Fig. 2C) (Table 1).

### Heterogeneity of CD34^+^ EB cells: CD34^+^CD4^−^ EB cells have lymphoid potential

**Figure 3:**
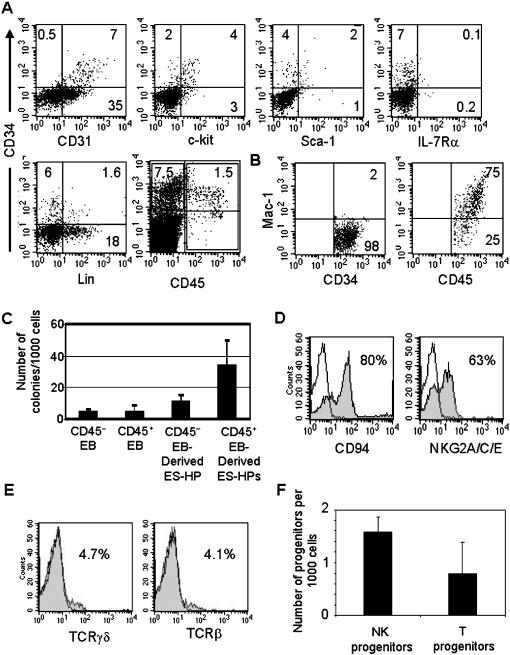
Characterization of CD34^+^CD45^+^ and CD34^+^CD45^−^ EB cells. (A) EBs were harvested on day 8 and single cell suspension was prepared. Cells were stained with anti-CD34 mAb together with mAb to indicated markers and analyzed by flow cytometry. Dead cells were gated out by propidium iodide staining. (B) CD34^+^CD45^−^ (left) and CD45^+^ (right) EB cells in (A) were gated, and analysed for the expression of Mac-1 by flow cytometry. Numbers indicate percentage of cells in quadrants or gates. (C) CD34^+^CD45^−^ and CD45^+^ EB cells were isolated by FACS sorting and cultured to generate ES-HPs. The sorted EB cells and the ES-HPs generated from them were plated for myeloid and erythroid colony formation as in Fig. 1F. (D) CD34^+^CD45^−^ EB cells were directly cultured on OP9 stroma with IL-2 and IL-15 for NK cell differentiation. Cells were harvested after 2 weeks, stained for NK markers and analyzed by flow cytometry as in Fig. 1B. (E) CD34^+^CD45^−^ EB cells were directly cultured onto OP9-DL1 stroma with proper cytokines for T cell differentiation for 3 weeks. Expression of T cell markers was analyzed by flow cytometry as in Fig. 1E. (F) Limiting numbers (100, 300 and 1,000 cells per well) of CD34^+^CD45^−^ EB cells were sorted into each well of 96 well plates. For NK progenitor assays, wells contained OP9 stroma, IL-2, and IL-15. For T progenitor assays, wells contained OP9-DL1 cells, IL-7 and Flt3-L as described in materials and methods. NK cells were detected by granzyme B RT-PCR and T cells detected by TCRγ RT-PCR followed by southern blotting. The number of progenitors in 1000 plated cells was calculated as in Fig. 2C.

The above results showed that the ES-HP population was very heterogeneous, containing hematopoietic and possibly non-hematopoietic (e.g. endothelial) cells, and the frequency of NK progenitors among ES-HPs was low. To determine whether this is due to heterogeneity of CD34^+^ EB cell population that gave rise to ES-HPs, we analyzed the expression of various hematopoietic cell markers on CD34^+^ EB cells (Fig. 3A). Large fractions of CD34^+^ cells expressed the hematopoietic progenitor-associated markers c-kit (65%) and Sca-1 (35%), and almost all expressed CD31, a marker for endothelial cells [21] and erythroid progenitors [22]. No significant levels of IL-7Rα (CD127) and mature hematopoietic lineage markers, including Mac-1, Gr-1, Ter119, B220, CD19, CD3 and CD8 were detected on CD34^+^ EB cells. Approximately 15–25% of CD34^+^ EB cells expressed the pan-leukocyte marker CD45.

We fractionated CD34^+^ EB cells into CD45^+^ and CD45^−^ subsets by FACS sorting and analyzed for myeloid/erythroid and lymphoid potentials. Both populations contained comparable frequencies of myeloid/erythroid colony forming cells (Fig. 3C). When they were cultured on OP9 stroma in the presence of SCF, Flt3-L, IL-7 and IL-6 for 7 days to generate ES-HPs and examined for the myeloid potential, the frequency of myeloid/erythroid colony forming cells in CD45^+^ EB-derived ES-HPs was significantly higher than that of CD34^+^CD45^−^ EB-derived cells. However, CD45^+^ EB cells or those cultured for ES-HP generation did not differentiate into NK, T or B cells upon co-culturing with OP9 or OP9-DL1 in the presence of appropriate cytokines (see materials and methods). In contrast, CD34^+^CD45^−^ EB cells cultured under the same conditions differentiated into NK and T cells (Fig. 3D, E). Limiting dilution analysis showed that on average 1 in 633 CD34^+^CD45^−^ EB cells differentiated into NK cells, while frequency of T progenitors was 1 in 1266 (Fig. 3F) (Table 1). No NK or T cell progenitors were detected among CD45^+^ EB cells by limiting dilution analysis (data not shown). These results indicate that lymphoid potential is contained in the CD45^−^CD34^+^ EB cell population whereas CD45^+^ EB cells have erythroid/myeloid but not lymphoid potential. Analysis of Mac-1 expression on CD34^+^ EB cells showed that in contrast to CD45^−^ subset, CD45^+^ subsets are Mac-1^+^, suggesting that the latter are committed myeloid precursors (Fig. 3B).

### NK cell progenitors among ES-HPs derived from CD34^+^CD45^−^ EB cells

**Figure 4:**
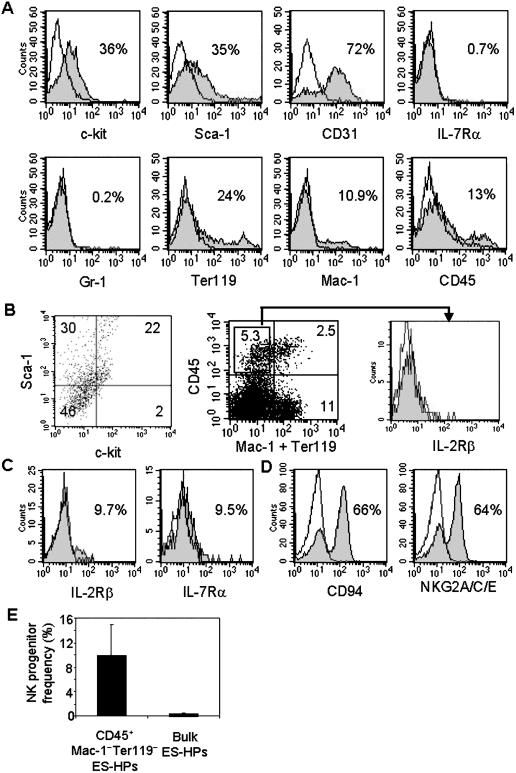
Characterization of ES-HP cells derived from CD34^+^CD45^−^ EBs for surface markers and NK potential. (A) ES-HP cells derived from CD34^+^CD45^−^ EB cells were stained with mAbs to indicated markers and analyzed by flow cytometry as in Fig. 2A. (B) ES-HP cells were co-stained with anti-CD45, c-kit and Sca-1 mAbs. Cells were then gated on CD45^+^ and analyzed for the expression of c-kit and Sca-1 (left panel). ES-HP cells were stained with a combination of purified anti-CD45.2 mAb followed by Alexa Flour 647-goat anti-mouse IgG, and biotinylated anti-Mac-1 and Ter119 mAbs followed by streptavidin-PE and analyzed by flow cytometer (middle panel). CD45^+^Mac-1^−^Ter119^−^ cells were gated and analyzed for the expression of IL-2Rβ (right panel). (C) CD45^+^Lin^−^ ES-HPs were sorted and cultured on OP9 stroma with IL-2 and IL-15. After 3 days, cells were harvested and subjected to flow cytometry analysis after staining with anti-IL-2Rβ and anti-IL-7Rα. (D) Sorted CD45^+^Lin^−^ ES-HPs were cultured in NK culture (described in materials and methods) for one week and analysed for the expression of NK markers. (E) The bulk and sorted CD45^+^Lin^−^ ES-HP cells were analyzed for NK progenitor frequency by limiting dilution cultures as in Fig. 2C. For the former 30, 100 and 300 cells per well and for the latter 3, 10, and 30 cells per well were plated. The results are mean ± SD of three independent experiments.

The above results showed CD34^+^CD45^−^ EB cells have lymphoid potentials but the frequencies of NK and T cell progenitors were rather low. Therefore, we generated ES-HPs from CD34^+^CD45^−^ EB cells and analyzed them for cell surface marker expression and lymphoid differentiation potentials. Flow cytometric analysis showed that the cells generated in vitro from CD34^+^CD45^−^ EB cells were again heterogeneous and included large fractions expressing c-kit, Sca-1, and CD31 whereas IL-7R was almost undetectable. Cells expressing the erythroid marker Ter119 and the myeloid cell marker Mac-1 (CD11b, CD18) were also detected (Fig. 4A). Only 13% of the cells were CD45^+^ (Fig. 4A), which included cells co-expressing c-kit and Sca-1 and those expressing Mac-1 and/or Ter119 (Fig. 4B).

NK potential of ES-HPs generated from CD34^+^CD45^−^ EB cells was first tested by bulk cultures. NK cells expressing CD94 and NKG2A/C/E were readily generated in cultures on OP9 stroma (data not shown). Limiting dilution analysis showed that NK progenitors are highly enriched in the CD45^+^Lin^−^ (Mac-1^−^Ter119^−^) subset of ES-HPs. In three experiments, the frequency of NK progenitors in this population was on average about 10% (1 in 10), ranging from 14% (1 in 7) to 4% (1 in 24) in three experiments (Fig. 4E), which is comparable to the frequency of NK progenitors in the BM NKP (Lin^−^CD122^+^) population [2], whereas the NK progenitor frequency of the bulk ES-HP population was on average 0.4% (1 in 230) (Table 1). As the CD45^+^Lin^−^ subset is about 5% of the total ES-HP population, most NK progenitors seemed enriched in this subset of ES-HPs.

However, CD45^+^Lin^−^ ES-HPs were different from BM-derived NKPs, as they do not express detectable level of IL-2Rβ (CD122) (Fig. 4B, right panel). When CD45^+^Lin^−^ ES-HPs were sorted and cultured with IL-2 and IL-15 for three days, IL-2Rβ was detected on the cultured cells (Fig. 4C). CD45^+^Lin^−^ ES-HP cells became mature CD94^+^ and NKG2A/C/E^+^ NK cells after one week in the NK differentiation culture (Fig. 4D).

### ES-HPs derived from CD34^+^CD45^−^ EB cells have T and B cell potentials

**Figure 5:**
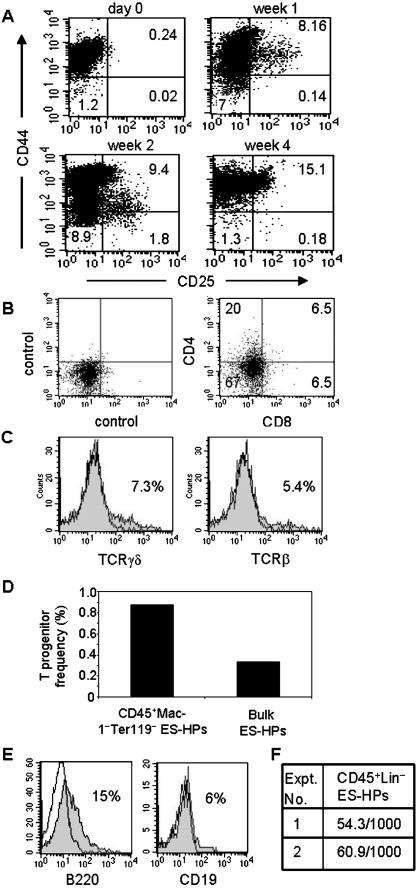
T, B and myeloid potential of ES-HPs derived from CD34^+^CD45^−^ EB cells. (A) CD34^+^CD45^−^ EB cells were sorted and cultured with OP9 and cytokines to generate ES-HPs. They were then cultured on OP9-DL1 stroma with cytokines for T cell differentiation. At different time points, cells were harvested and stained for CD44 and CD25 and analyzed by flow cytometer. Dead cells were stained with propidium iodide and OP9-DL1 cells expressing green fluorescent protein were gated out. The numbers show the percentages of cells in the quadrants. (B) ES-HP cells were cultured for T cell differentiation for 8 days with 5 ng/ml IL-7 as in (A). On day 8, IL-7 concentration was reduced to 1 ng/ml and cells were cultured for an additional 6 days. Cells were harvested and analyzed by flow cytometer for CD4 and CD8 expression. The numbers indicate the percentages of cells in the quadrants. (C) ES-HP cells were cultured for T cell differentiation with 5 ng/ml IL-7 for 2 weeks and an additional 4 days with 1 ng/ml IL-7, stained for TCRγδ and TCRβ and analyzed by flow cytometer. The numbers show the percentages of cells positively stained with the test antibodies (filled histogram) over control antibody (open histogram). (D) ES-HPs were generated from CD34^+^CD45^−^ EB cells as in (A). The bulk and sorted CD45^+^Lin^−^ ES-HP cells were analyzed for T progenitor frequency by limiting dilution cultures as in Fig. 3F, except that 30, 100, 300 and 900 cells per well for bulk and 10, 30 and 100 cells per well for CD45^+^Lin^−^ cells were plated. The results are average of two independent experiments. (E) CD45^+^Lin^−^ ES-HPs were sorted and cultured on OP9 cells with IL-7 and Flt3-L for B cell generation. After one week, cells were harvested and analysed for the expression of B220 and CD19 with flow cytometer. (F) One thousand sorted CD45^+^Lin^−^ ES-HPs were transferred into myeloid differentiation media as in Fig. 1F. The number of myeloid and erythroid colonies were scored.

To test whether ES-HPs derived from CD34^+^CD45^−^ EB cells have T cell differentiation potential, they were cultured on OP9-DL1 stromal cells in the presence of IL-7 and Flt3-L. We first analyzed the expression of CD44 and CD25 at different time points during the culture (Fig. 5A) as these markers are commonly used to define the early steps of immature double negative (DN, CD4^−^CD8^−^) thymocyte differentiation [23]. The cells initially resembled the most immature thymocyte population DN1 (CD4^−^CD8^−^CD44^+^CD25^−^). After one week of culture, some (∼8%) of the cells acquired CD25 and resembled DN2 (CD4^−^CD8^−^CD44^+^CD25^+^) thymocytes. After two weeks, the level of CD44 on DN2-like cells decreased and they resembled DN3 (CD4^−^CD8^−^CD44^−^CD25^+^) thymocytes. Prolonged culture did not induce a further differentiation into the DN4 (CD4^−^CD8^−^CD44^−^CD25^−^) population. Although CD44^−^CD25^−^ cells were detected early in the cultures (week one and two), they seemed to directly derive from the DN1-like cells without going through the DN2 and DN3 stages. Very few cells expressing CD4, CD8 or CD3 were generated in this culture (data not shown). It appeared that T cell development from ES-HPs was impaired at the transition from DN3 to DN4. This may be due to a high dose of IL-7, which has been reported to block differentiation of adult progenitors into T cells [24], [25]. Therefore, after 8–14 days of culture, the IL-7 concentration was reduced to 1 ng/ml, and the cells were cultured for an additional 4–6 days with OP9-DL1. Cells expressing CD4, CD8 and the TCR were detected in the low-dose IL-7 culture (Fig. 5B, C). However, the expression levels of CD4 and CD8 were significantly lower than those on thymocytes (data not shown), and very few double positive (CD4^+^CD8^+^) and single positive (CD4^+^CD8^−^ and CD4^−^CD8^+^) cells were generated.

Limiting dilution analysis showed that the frequency of T cell progenitors was approximately 0.3% (1 in 297) in the bulk ES-HP population and 0.9% (1 in 115) in the CD45^+^Lin^−^ subset of ES-HPs (Fig. 5D). Thus, T cell progenitors were enriched in the CD45^+^Lin^−^ subset, which contained most of the NK progenitors. However, the CD45^+^Lin^−^ subset was about 5% of the bulk ES-HPs while the enrichment of T cell progenitors was only about three fold, suggesting that T cell progenitors were also contained in other subsets of ES-HPs.

To test the B cell potential of ES-HPs, CD45^+^Lin^−^ ES-HPs were sorted and cultured on OP9 with IL-7 and Flt3-L for one week. B cells were detected in the culture by the expression of B220 and CD19 (Fig. 5E). Therefore, CD45^+^Lin^−^ ES-HPs contained B cell potential. To test whether these cells also have myeloid differentiation potential, sorted CD45^+^Lin^−^ ES-HPs were transferred into methylcellulose for myeloid colony formation. On average, 5.7% of CD45^+^Lin^−^ ES-HPs formed erythro/myeloid colonies (Fig. 5F). These results indicate that ES-HPs have NK, T, B and myeloid/erythroid potentials.

## Discussion

We have characterized NK cell progenitors at various stages of development from ES cells in vitro. The most immature progenitors with NK cell potential are found among EB cells expressing CD34 but not the pan-leukocyte marker CD45. When cultured on OP9 stroma in the presence of IL-7, IL-6, Flt3-L and SCF, CD34^+^CD45^−^ EB cells differentiate into a heterogeneous cell population, which we termed ES-HP (ES cell-derived hematopoietic progenitors). Among ES-HPs, NK cell progenitors are highly enriched in a subpopulation expressing CD45 but not the mature erythroid and myeloid cell lineage markers Ter119 and Mac-1. Thus, CD34^+^CD45^−^ immature hematopoietic progenitors in EBs differentiate into more mature CD45^+^ NK progenitors in this culture. Since CD34^+^CD45^−^ EB cells are heterogeneous, the relationship between NK progenitors in the EB cell population and those among ES-HPs is still unclear. However, the difference in CD45 expression indicates that NK progenitors among ES-HPs are not products of simple amplification of NK progenitors in EB but rather they are differentiation products derived from immature hematopoietic progenitors in EBs.

The overall ES-HP cell population is rather heterogeneous and contains myeloid and erythroid cells at various stages of development as well as CD45^−^Lin^−^ cells, which mostly includes immature hematopoietic progenitors (unpublished data, 2007). Most, if not all, NK progenitors are in the CD45^+^Lin^−^ (Ter119^−^Mac-1^−^) subset. The frequency of NK progenitors in this subset, as determined by limiting dilution assay, is about 10%, which is comparable to that of adult mouse BM NKPs defined by CD122^+^Lin^−^ [2]. However, there are some differences between NKP in BM and those in ES-HPs. Most notably, BM NKPs express CD122 [2] whereas it is undetectable on those among ES-HPs (Fig. 4B, right panel). It is likely that NK progenitors at the ES-HP stage are more immature than BM NKPs. CD122^−^ NK progenitors, which mature into CD122^+^ progenitors, have been found in human lymph nodes as well [26]. As expected, NK progenitors in the ES-HP population rapidly acquire CD122 during their maturation to NK cells (Fig. 4C).

The frequency of T progenitors in bulk ES-HPs is significantly higher than that among EBs (1 in 297 versus 1 in 1266, respectively), indicating a T cell developmental intermediate is generated in ES-HP culture. However, the enrichment of T cell progenitors in CD45^+^Lin^−^ ES-HP fraction compared to bulk population was much less than that of NK progenitors (three fold versus 25 fold). Nevertheless, these results show that T progenitors co-exist with NK progenitors in the same cell population. It is still unclear whether ES-HPs include bipotent T/NK progenitors. Both T and NK cells are generated in bulk cultures to induce differentiation of CD45^+^Lin^−^ ES-HPs into T cells. However, the results with limiting dilution analysis show that the frequency of T cell progenitors among CD45^+^Lin^−^ ES-HPs is low (less than 1%). Our attempt to determine the frequency of progenitors that give rise to both NK and T cells by limiting dilution assays on OP9-DL1 cells have resulted in rather inconsistent results (data not shown). One of the difficulties in determining T cell progenitor frequency in this study is that most T cell progenitors do not differentiate into mature T cells expressing the TCR, CD3, CD4 or CD8 in our cultures as their differentiation is blocked at the DN2 stage before TCRγ gene rearrangement, which was used to detect T cells in the limiting dilution analysis. Thus, the frequency of T cell progenitors is likely very much underestimated in our study, making it difficult to detect bipotent T/NK progenitors. Nevertheless, our study has shown that CD45^+^Lin^−^ ES-HPs include both NK and T cell progenitors. We also showed that CD45^+^Lin^−^ ES-HPs have B cell and myeloid potentials. Thus CD45^+^Lin^−^ ES-HPs likely comprise of multiple committed progenitors. However, it is also possible that CD45^+^Lin^−^ ES-HPs include multi-potent hematopoietic progenitors which are downstream progeny of CD45^−^ progenitors.

Our study has also shown that CD34^+^ EB cells are rather heterogeneous and include both CD45^+^ and CD45^−^ cells. Although the relationship between these two subsets of CD34^+^ EB cells is still unclear, the former includes erythroid/myeloid colony forming cells but no lymphoid progenitors whereas the latter has both myeloid/erythroid and lymphoid potentials. Since CD34^+^CD45^+^ EB cells express Mac-1 (Fig. 3B) , it is possible that these cells are myeloid precursors that have derived from CD45^−^ EB cells generated earlier in the EBs. CD45^+^ macrophage precursors have been described during early embryonic development in aorta-gonad-mesonephros (AGM) region [27]. On the other hand, CD45^−^ EB cells resemble precursors found in paraaortic splanchnopleura (P-Sp) and AGM region which generate lymphocytes and also lack CD45 [27], [28]. Through an in vitro differentiation system, for the first time, we have demonstrated a differentiation pathway in which these cells acquire CD45 during ES-HP stage before subsequent differentiation to individual hematopoietic lineages. It will be interesting to compare the reconstitution capacity of CD45^−^ EBs and that of CD45^+^Lin^−^ ES-HPs. While EB cells have been shown to reconstitute adult BM only by overexpression of *HoxB4* [29] and/or *Cdx4* [30], CD45^+^ ES-HPs may be developmentally closer to adult HSCs and give rise to multilineage engraftment without a need for genetic modifications.
